# Usefulness of ^18^F-FDG PET/CT in Cutaneous Melanoma Patients with Negative Sentinel Lymph Nodes and High Clark Levels

**DOI:** 10.4274/mirt.70783

**Published:** 2018-06-07

**Authors:** Özge Vural Topuz, Fatma Arzu Görtan, Zübeyde Rana Kaya Döner, Çetin Önsel, Haluk Burçak Sayman

**Affiliations:** 1 University of Health Sciences, Okmeydanı Training and Research Hospital, Clinic of Nuclear Medicine, İstanbul, Turkey; 2 University of Health Sciences, Ankara Atatürk Training and Research Hospital, Clinic of Nuclear Medicine, Ankara, Turkey; 3 Kahramanmaraş Necip Fazıl City Hospital, Clinic of Nuclear Medicine, Kahramanmaraş, Turkey; 4 İstanbul University Cerrahpaşa Faculty of Medicine, Department of Nuclear Medicine, İstanbul, Turkey

**Keywords:** 18F-FDG, melanoma, sentinel lymph node biopsy

## Abstract

**Objective:**

We investigated the utility of PET/CT in cutaneous melanoma (CM) patients with pathological negative sentinel lymph nodes (SLN), within the first year.

**Methods:**

The results of PET/CTs and SLN biopsy (SLNB) in 65 patients (39 male and 26 female, mean age 53.8) with a PET/CT in the first postoperative year were evaluated. Within this cohort, the utility of early PET/CT imaging was assessed in patients with negative SLNB. McNemar test was used to compare PET/CT findings with SLNB results.

**Results:**

Out of 43 patients with pathologically positive SLNs, 23 patients (53.5%) had positive and 20 patients (46.5%) had negative findings on PET/CT within the first postoperative year. On the other hand, PET/CT results of 22 patients with negative SLNBs were found to be negative in 19 patients (86.4%) and positive in 3 patients (13.6%). For the patients with negative SLNB results, the detection rate of distant metastasis with PET/CT was significantly lower (p<0.001) than that in patients with positive SLNBs.

**Conclusion:**

Our results showed that ^18^F-FDG PET/CT will most likely (86.4%) be negative during the first postoperative year in patients with a negative SLNB. Therefore, it is concluded that this modality would not provide any significant clinical contribution within this time frame.

## Abstract (tr)

**Amaç:**

Çalışmamızda sentinel lenf nodu (SLN) patoloji sonucu negatif olan malign melanom (MM) tanılı hastalarda PET/BT’nin ilk bir yıl içerisindeki yararını araştırdık.

**Yöntem:**

Ameliyat sonrası ilk bir sene içerisinde PET/BT çekimi yapılan 65 hastanın (39 erkek; 26 kadın, yaş ortalaması: 53,8) PET/BT çekimleri ve SLN biyopsi sonuçları değerlendirildi. Bunların içerisinden SLN biyopsi sonucu negatif olan hastalarda erken dönemde PET/BT görüntülemenin gerekliliği araştırıldı. McNemar testi, pozitif veya negatif PET/BT sonuçlarını pozitif ve negatif SLNB sonuçları ile karşılaştırmak için kullanıldı.

**Bulgular:**

SLNB sonuçları pozitif olan 43 hastanın postoperatif 1. yılda yapılan PET/BT çekimlerinin sonuçları 23 hastada (%53,5) pozitif, 20 hastada (%46,5) ise negatif olarak bulundu. Diğer taraftan SLN biyopsi sonucu negatif olan 22 hastanın postoperatif 1. yılda yapılan PET/BT çekimlerinin sonuçları 19 hastada (%86,4) negatif, 3 hastada (%13,6) ise pozitif olarak bulundu. SLN biyopsi sonucu negatif olan hastalar için postoperatif 1. yılda yapılan PET/BT ile uzak metastaz saptanma oranı SLN biyopsi sonucu pozitif olan hastalardan anlamlı olarak daha düşük (p<0,001) bulundu.

**Sonuç:**

Çalışmamızda, SLN biyopsi sonucu negatif olan hastalarda postoperatif ilk bir yılda yapılan ^18^F-FDG PET/BT çekimlerinin sonuçlarının büyük bir olasılıkla (olguların %86,4’ünde) negatif olması nedeniyle bu hasta grubunda bu periyotta yapılan ^18^F-FDG PET/BT çekimlerinin önemli bir klinik katkı sağlamayacakları sonucuna varılmıştır.

## Introduction

PET/CT is introduced as an important scintigraphic imaging method for both the diagnosis and restaging of maligant diseases as well as the evaluation of treatment outcomes. Although it is a valuable method, it is a costly procedure with considerable radiation exposure to tissues. That is why, it might be feasible to avoid PET/CT scanning in unnecessary conditions in terms of overall patient health.

The use of ^18^F-FDG PET/CT for both staging and follow-up of patients with cutaneous melanoma (CM), which is a disease that threatens life and spreads easily with lymphatic metastases, has been previously reviewed by clinicians (1,2,3,4). 

Sentinel lymph node (SLN) biopsy (SLNB) has an important role in disease staging of these patients. Although PET/CT has been proven to be useful in the staging of CM, it should only be used in patients when truly indicated. The utility of PET/CT to detect systemic recurrences during follow-up has yet to be investigated. SLNB results might have clues to determine the exact timing of this examination.

The purpose of this study was to evaluate the necessity and utility of PET/CT imaging that was performed within the first year in patients with a diagnosis of CM, whose SLN(s) have previously been biopsied by either blue dye injection or lymphoscintigraphy and had negative results for metastasis.

## Materials and Methods

Between January 2005 and December 2010, the results of 425 PET/CT examinations of 360 patients, who have been diagnosed histopathologically as CM and referred to our department for restaging by PET/CT imaging, were evaluated retrospectively. Within 360 patients, 96 patients who had postoperative pathology results of their SLNBs were included in the study. Patients had been selected for SLNB according to NCCN guidelines.

Accordingly, patients with melanoma greater than 1 mm in depth along with those between 0.76 mm and 1.0 mm in thickness had been considered for SLND if they had positive deep margins, lymphovascular invasion, age less than 40 years, significant vertical growth phase, increased mitotic rate, and Clark’s level IV or higher (5).

In order to reach the maximum number of examinations possible within the same interval, only the results of 65 patient’s [39 male and 26 female, mean age=53.8 (minimum=27; maximum=85)] PET/CT studies that have been performed in the first postoperative year were evaluated as well as their SLNB pathology results.

Within this cohort, 22 patients with negative SLNB results were determined and the utility of early PET/CT imaging in this subgroup was further evaluated.

The lesions were located mostly at the feet (n=14), back (n=9) and arms (n=7). The rest of the lesions were found on sites such as the scalp, ears, cheeks, abdomen, legs and hands in decreasing order. According to histopathologic subtypes, most patients were diagnosed with superficial spreading type of CM followed by nodular and acrolentiginous types in decreasing frequency (Table 1).

The Breslow thickness of the detected lesions varied between 0.6-17.0 mm. Thus, the Clarks levels were between 3 and 5.

PET/CTs were performed at our department by a high-resolution PET scanner (Siemens Biograph LSO HI-RES PET/CT, Illinois, USA) integrated 6 section multidetectors (for attenuation correction and location).

The patients fasted for at least 4 hours and their finger-tip blood glucose levels were under 150 ng/mL. They rested to obtain complete biodistribution of ^18^F-FDG in the body for about 1 hour after an intravenous injection of 13-15 mCi of ^18^F-FDG. After micturition they were taken into the imaging suite and were laid on the scanner bed in the supine position.

Following a low-dose CT scan without iv. radiological contrast agent injection PET scanning was performed, covering all areas of the body. Then, CT and PET data were reconstructed by iterative methods after performing attenuation correction for PET images by using tissue attenuation map derived from corresponding CT data. Fusion images of PET and CT at three spatial planes (axial, coronal, and sagittal) approximately 0.5 cm in thickness cross-sectional images as well as maximum intensity projection (MIP) images were displayed and evaluated by an experienced nuclear medicine specialist on a high-resolution monitor. When it is deemed necessary, PET images without attenuation correction were interpreted by the examiner. In the interpretation, excluding the physiological FDG uptake, the foci showing more intensive involvement as compared to the environmental background activity were evaluated as metastasis and the result was expressed as a positive PET/CT finding. In addition, a semi-quantitative parameter indicating the rate between the average activity in the body and intensity of activity in the selected area i.e. “maximum standard uptake value (SUV_max_)” was also used for pathological uptake evaluation.

Preoperative lymphoscintigraphy by intracutaneous injection of 0.5 mCi Tc-99m-labelled albumin nano-colloids around the tumor or the tumor’s excision scar was combined with the intraoperative use of a hand-held gamma probe and patent blue dye mapping technique in order to identify and harvest the SLN.

### Statistical Analysis

McNemar test was used in order to compare the positive or negative PET/CT results of the study group with the positive and negative results of their SLNBs. Statistical results were expressed as percent and frequency. Any “p” value less than 0.05 was accepted as statistically significant.

### Ethics Committee Approval and Informed Consent

This study was approved by the İstanbul University Cerrahpaşa Medical Faculty Ethics Committee (date: 12 July 2010, file number: 20697) and signed written informed consent was obtained from all research subjects.

## Results

Study patients were divided into 7 groups according to the time interval between the surgery of the primary lesion and the initial PET/CT (Table 2). Some patients in these groups had more than one PET/CT scans during their course of follow-up.

In the first postoperative year, PET/CT results were positive in 26 (40%) and negative in 39 (60%). Of the same patients, SLNB results were positive in 43 (66.2%) and negative in 22 (33.8%) (Table 3).

**Figure 1:**
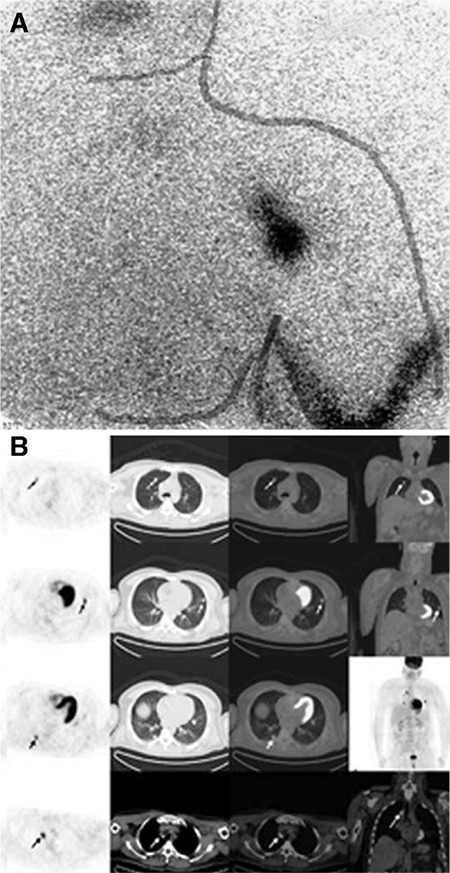
Preoperative lymphoscintigraphy image of a patient with a positive axillary sentinel lymph nodes biopsy (A) along with metastatic lun nodules and mediastinal lymph nodes of the same patient in PET/CT scan performed within the 1^st^ postoperative year (B)

**Figure 2:**
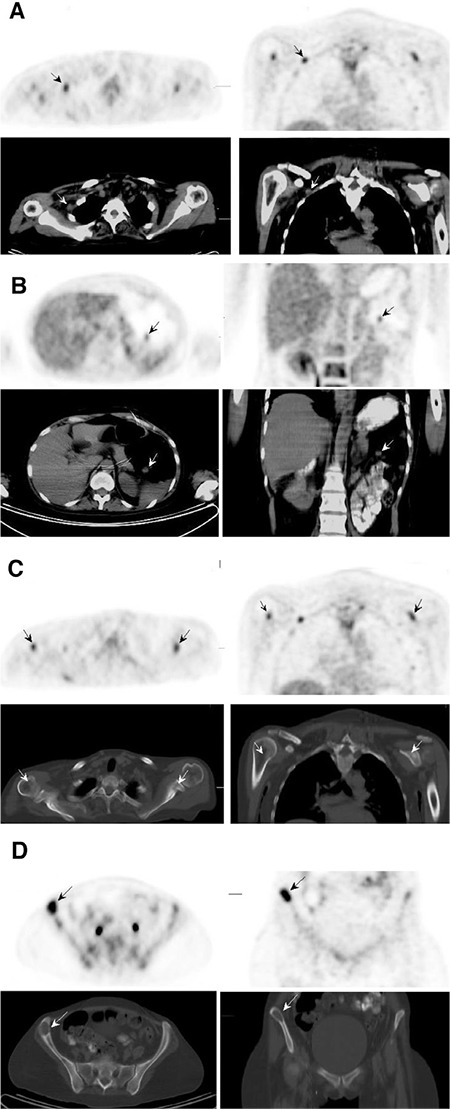
PET/CT images performed within the 1^st^ postoperative year of a patient with primary tumor localized at the foot with a negative sentinel lymph nodes biopsy, showing right axillary lymph node metastasis (A), mesenteric metastasis (B), and bone metastasis (C and D)

Within the first postoperative year, PET/CT results of 43 patients with a positive SLNB were found to be also positive in 23 patients (53.5%) (Figure 1A, 1B) and negative in 20 patients (46.5%). On the other hand, PET/CT results of 22 patients with a negative SLNB were found to be also negative in 19 patients (86.4%) and positive in 3 patients (13.6%). One of the three patients with a negative SLNB and positive PET/CT finding was a 47-year-old female patient with superficial spreading melanoma localized in the abdomen, the tumor being Clark level III with a thickness of 0.6 mm without ulceration. The second was a 76-year-old male patient with malignant melanoma of the back while the third patient was a 60-year-old male patient with the primary tumor localized at the foot showing a histopathology of Clark level V subungual melanoma, tumor thickness of 0.6 mm with ulceration (Figure 2A, 2B, 2C, 2D).

For the patients with negative SLNB results, the detection rate of distant metastasis with PET/CT was significantly lower (p<0.001) than that in patients with positive SLNB results (Table 4).

## Discussion

In a study on the routine use of ^18^F-FDG PET in patients with early stage CM and positive SLNB results by Horn et al. (6), ^18^F-FDG PET was reported to be positive in 27% of these cases. However, only 12% of these have been verified as true CM metastases. According to this result, even in case of positive SLNB results, FDG PET imaging did not have a clinically important impact for staging. On the contrary, our results demonstrated that 53.5% of patients with a positive SLNB also had a positive PET/CT scan in terms of metastatic lesions, which indicates that in approximately half of the study patients any possibly metastatic lesion could be detected by PET/CT. Therefore, PET/CT is a feasible method in these patients to detect possible distant metastatic lesions.

Constantinidou et al. (7) have reported that PET scan was positive on two patients none of which was related to melanoma out of 30 patients who were referred for PET/CT within 100 days postoperatively with positive SLNB results, and the value of PET was determined to be limited for staging, although they have indicated that FDG PET was a highly sensitive and specific method in identifying distant metastases.

The positive SLNB rate is reported as approximately 20% in most studies on CM patients. In our study group, this rate was determined to be much higher (66.2%) which may be attributed to the significantly advanced disease stage of our patients, having Clark levels mostly greater than 4.

Wagner et al. (8) performed ^18^F-FDG PET or PET/CT scans in 46 CM patients in order to determine the rate of distant metastasis, within 6 weeks after positive SLNBs. Of these 46 patients, 6 had an inconclusive PET/CT. The distant metastases were detected in 5 (12.5%) of the remaining 40 patients with negative PET results by other methods within 12 months. In this study, FDG PET was found inadequate in detecting distant metastases during initial staging of these patients with positive SLNB results. These results may be due to the lack of macroscopic metastatic disease detectable by PET scan performed within six weeks after positive SLNBs.

In other similar studies, PET/CT was found to be ineffective in early stages of CM even in patients with positive SLNBs. Actually, it is thought that this insufficiency could be more pronounced in t patients with negative SLNBs, which was the case in our study. In line with these findings, our results indicated that 86.4% of the ^18^F-FDG PET/CTs performed in the first postoperative year in patients with negative SLNB results were also negative.

In another study evaluating the accuracy of SLNB and ^18^F-FDG PET imaging for determining early lymph node metastasis by Libberecht et al. (9), preoperative PET imaging and SLNB were performed in five patients diagnosed with CM over 1 mm Breslow thickness and without clinically detected lymph node metastases and recurrence. In this study, no lymph node involvement or distant metastasis were detected by PET while micrometastasis was histologically detected in the SLN in 2 of the 5 patients (40%) included in the study during the 10 month study period. The false negative finding of lymph node metastasis by PET in this small group of patients indicated that PET had a limited role for this group of patients without palpable lymph nodes. Accordingly, even when there was micrometastasis in the SLN(s), it was determined that the probability of distant organ metastasis detection by PET performed within 10 months would be low. In other words, keeping the limited number of patients in mind, ^18^F-FDG PET together with SLNB was found to be negative in 60% of their patients.

In a retrospective study of 61 patients with early stage CM, Klode et al. (10) reported that only 1 of 17 metastatic SLNs was detected in 14 patients (23%) by preoperative ^18^F-FDG PET/CT. In that study, the sensitivity and the negative predictive value of ^18^F-FDG PET as a diagnostic test was calculated as 5.9% and 78%, respectively. It was concluded that ^18^F-FDG PET was not an appropriate method for evaluation of early regional lymphatic metastases and that ^18^F-FDG PET would be more applicable for AJCC stage III-IV CM patients.

Some of the aforementioned studies have reported that ^18^F-FDG PET imaging was not sensitive in detecting SLN metastases. For example, in a study on patients diagnosed with early-stage CM Wagner et al. (8) reported the true positivity rate of FDG PET as 11%. We could not make a similar evaluation since we did not evaluate ^18^F-FDG uptake particularly for SLN(s). Moreover, in that study distant metastasis could be detected only in 12.5% of patients by early ^18^F-FDG PET imaging. According to our study, recurrence or distant metastasis could be demonstrated in 53.5% of cases with a positive SLNB within the first year by ^18^F-FDG PET/CT.

It was an interesting finding that in 3 patients whose SLN was negative, the PET/CT results were positive. Unfortunately, we could not depict any underlying reason to explain it. There was diversity in terms of their age, gender, site and type of lesions.

### Study Limitations

The main limitation of our study was not to perform histopathologic verification for foci of ^18^F-FDG uptake in the study group. However, the patients were followed-up for long periods and secondary malignancy was not detected in any patients, that could have been interpreted as a false positive metastatic disease for CM.

## Conclusion

The existing published articles have focused on the utility of ^18^F-FDG PET or PET/CT to demonstrate distant metastases in patients with positive SLNBs, and this imaging method was not recommended at early stages of this disease for this particular purpose. Keeping that in mind, we tried to understand if this method is useful in the initial workup of CM patients with negative SLNBs.

In conclusion, ^18^F-FDG PET/CT performed within the first year after excision of the primary lesion in patients with CM was positive in 53.5% of patients with a positive SLNB, and was negative in 86.4% of patients with a negative SLNB.

It is well known that, SLNB does not provide direct information on the presence or absence of distant metastases (6). Nevertheless, our study also demonstrated that the presence of distant metastases within the first year was more than two-fold higher in patients with regional SLN involvement as compared to patients without SLN metastasis.

The results of our study showed that results of ^18^F-FDG PET/CT performed during the first year follow-up of patients histologically diagnosed with CM and had negative SLNB had a good correlation. Accordingly, ^18^F-FDG PET/CT was found to be negative during the first year follow-up of patients with a negative SLNB. Therefore, it is concluded that ^18^F-FDG PET/CT scanning will not provide any significant clinical contribution. The results also suggested that both local and distant metastases could be demonstrated by ^18^F-FDG PET/CT in more than half of the patients with a positive SLNB result and that PET/CT scanning was important in terms of re-staging.

## Figures and Tables

**Table 1:**
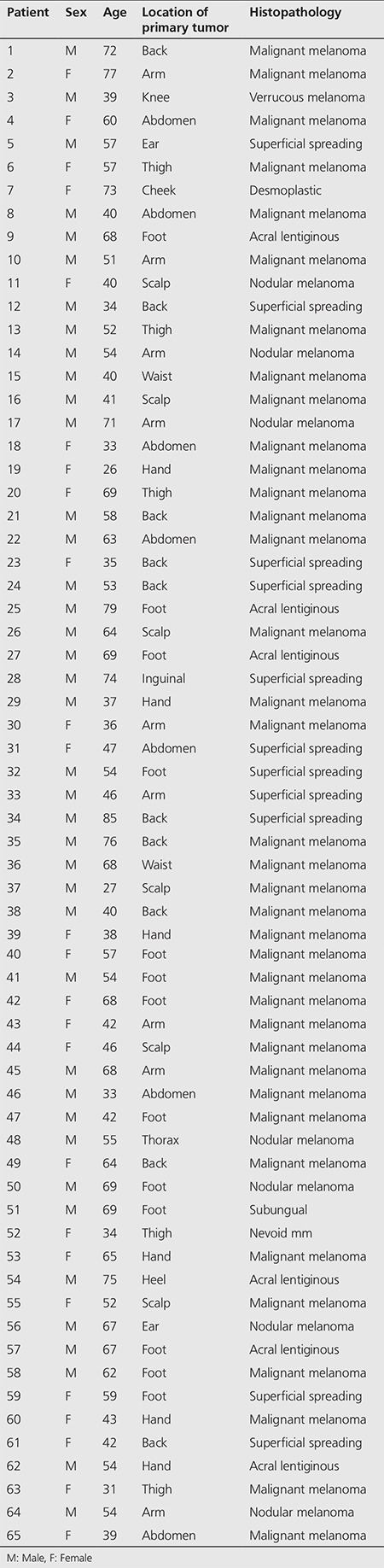
Patient characteristics

**Table 2:**
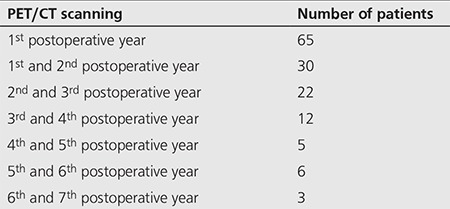
Patient distribution according to time of PET/CT scanning

**Table 3:**
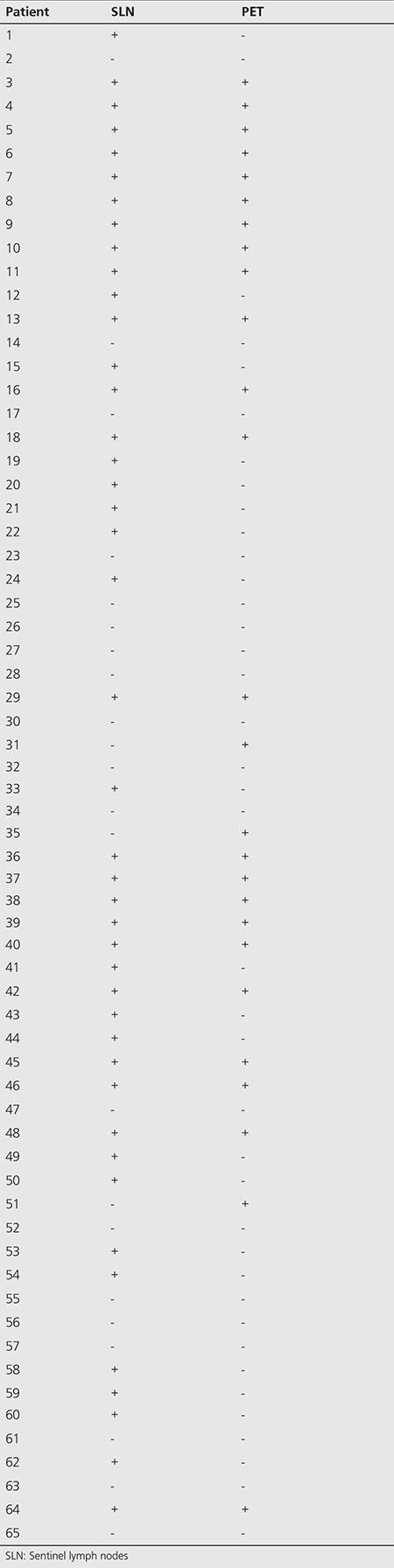
Sentinel lymph nodes biopsy results and PET/CT results of all patients

**Table 4:**
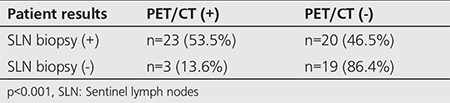
The correlation of sentinel lymph nodes biopsy and PET/CT results
